# Berberine ameliorates cellular senescence and extends the lifespan of mice via regulating p16 and cyclin protein expression

**DOI:** 10.1111/acel.13060

**Published:** 2019-11-26

**Authors:** Yao Dang, Yongpan An, Jinzhao He, Boyue Huang, Jie Zhu, Miaomiao Gao, Shun Zhang, Xin Wang, Baoxue Yang, Zhengwei Xie

**Affiliations:** ^1^ State Key Laboratory of Natural and Biomimetic Drugs Department of Pharmacology School of Basic Medical Sciences Peking University Beijing China; ^2^ Key Laboratory of Molecular Cardiovascular Sciences Ministry of Education Beijing China

**Keywords:** aging, berberine, cellular senescence, cyclin protein

## Abstract

Although aging and senescence have been extensively studied in the past few decades, however, there is lack of clinical treatment available for anti‐aging. This study presents the effects of berberine (BBR) on the aging process resulting in a promising extension of lifespan in model organisms. BBR extended the replicative lifespan, improved the morphology, and boosted rejuvenation markers of replicative senescence in human fetal lung diploid fibroblasts (2BS and WI38). BBR also rescued senescent cells with late population doubling (PD). Furthermore, the senescence‐associated β‐galactosidase (SA‐β‐gal)‐positive cell rates of late PD cells grown in the BBR‐containing medium were ~72% lower than those of control cells, and its morphology resembled that of young cells. Mechanistically, BBR improved cell growth and proliferation by promoting entry of cell cycles from the G_0_ or G_1_ phase to S/G_2_‐M phase. Most importantly, BBR extended the lifespan of chemotherapy‐treated mice and naturally aged mice by ~52% and ~16.49%, respectively. The residual lifespan of the naturally aged mice was extended by 80%, from 85.5 days to 154 days. The oral administration of BBR in mice resulted in significantly improved health span, fur density, and behavioral activity. Therefore, BBR may be an ideal candidate for the development of an anti‐aging medicine.

## Graphical abstract

A traditional Chinese medicine Berberine (BBR) extends lifespan at late stage and restores fitness and fur density in natural and accelerated aging mice. BBR down‐regulates p16 in senescent cells and ameliorates cellular senescence and senescence‐associated markers.

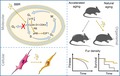

## 1 INTRODUCTION

Aging is characterized by a progressive loss of physiological integrity resulting in impaired function along with increased vulnerability to diseases and consequently death. This deterioration is the primary risk factor related to major human chronic noncommunicable diseases including cancer, diabetes, cardiovascular, and neurodegenerative diseases (Lopez‐Otin, Blasco, Partridge, Serrano, & Kroemer, 2013).

The discovery of the first long‐lived mutant strain in *Caenorhabditis elegans* (Son, Altintas, Kim, Kwon, & Lee, 2019) was a breakthrough in aging research. This finding aimed to find appropriate means to extend the lifespan of eukaryotic species, from single‐celled yeast to humans. Several approaches were demonstrated to prolong the lifespan both in vitro as well as in vivo, such as clearance of senescent cells (Baar et al., 2017), parabiosis (Villeda et al., 2014), and application of Yamanaka factors (Ocampo et al., 2016). However, it is difficult to translate these methods of extending the lifespan in humans into therapy. Oral drugs present a convenient medium for lifespan intervention. Previously, dasatinib + quercetin (Xu et al., 2018), fisetin (Yousefzadeh et al., 2018), metformin (Zakeri et al., 2019), rapamycin (Harrison et al., 2009), nicotinamide mono‐nucleotide (NMN) (Zhang et al., 2016), etc., were reported to extend lifespan in mice. However, more drugs are required to overcome the safety and cost issues.

Cellular senescence is one of the most important in vivo mechanisms related to aging. Senescent cells impair tissue function by irreparable cell damage resulting from acute stress or natural aging, consequently restricting the lifespan (Childs, Durik, Baker, & Deursen, 2015). Cellular senescence can be categorized into two groups. The replicative senescence, seen after approximately sixty rounds of cell division in cultures (Hayflick's limit) (Hayflick, 1965), results from the progressive erosion of telomeres following each division. This progressive erosion leads to telomere dysfunction and irreversible cell‐cycle arrest. The second category is defined as premature cellular senescence. It is unrelated to telomere shortening but is related to persistent cellular stress. Thus, replicative stress caused by oxidative DNA damage, activation of oncogenes, and loss of tumor suppressor genes also results in premature senescence. Furthermore, premature senescence includes irreversible impairment of tumor cell reproductive capability *via* chemotherapy or radiotherapy‐induced apoptosis which is defined as a drug or radiation‐induced senescence. The in vivo stress‐induced premature senescence of normal cells is considered to be a critical mechanism affecting organismal aging and longevity (Davalli, Mitic, Caporali, Lauriola, & D'Arca, 2016).

Berberine (BBR), a natural alkaloid found in *Coptis chinensis*, has a long history of medicinal use in both Ayurvedic and traditional Chinese medicine. It is commonly used as a dietary supplement for treating diarrhea. Furthermore, BBR possesses anti‐cancer (Ortiz, Lombardi, Tillhon, & Scovassi, 2014), anti‐inflammatory (Li et al., 2018), and anti‐neurodegenerative (Ahmed et al., 2015) properties. Although the biological properties of BBR are well‐documented (Cicero & Baggioni, 2016), there is little evidence of its role in anti‐aging processes. It was previously observed that BBR inhibited mTOR/S6 signaling concurrent with the reduction in the level of endogenous oxidants and constitutive DNA damage response (Zhao, Halicka, Li, & Darzynkiewicz, 2013). BBR also prolonged lifespan and improved viability of the wild‐type *Drosophila melanogaster* pupae, and the climbing activity of adult insects at higher temperatures is known to accelerate aging in wild‐type flies (Navrotskaya, Oxenkrug, Vorobyova, & Summergrad, 2014).

Thus, it was hypothesized that BBR, with its potential anti‐aging effects, could treat the senescence in aging cells. Yeast and human fetal lung diploid fibroblasts (2BS and WI38) were chosen as model systems to investigate the effects of BBR on anti‐aging in vitro, while naturally aged and chemo‐treated mice were used for in vivo studies.

## 2 MATERIALS AND METHODS

### 2.1 Berberine

Berberine was purchased from DESITE Biotechnology Co., Ltd (NO. DX0009), Chengdu, China. The average molecular weight was approximately 336.36 Da, as determined by high‐performance steric exclusion chromatography analysis. In our experiments, BBR was dissolved in 0.9% normal saline (NS) for the in vivo experiments.

### 2.2 Yeast growth conditions

Yeast was incubated on a standard liquid *SD* medium (1% yeast extract, 1% Bacto Peptone, 2% glucose) on a rotary shaker at 250 rpm at 30°C. For lifespan experiments, all strains were incubated overnight in a liquid culture (OD was kept below 0.8) and diluted 4 hr before acquisition so that cultures were in the logarithmic phase at the time of imaging.

### 2.3 Analysis of yeast replicative lifespan (RLS)

Mother cells were monitored for two days by repeated microscopic imaging as described (Xie et al., 2012). Micro‐posts integrated into the microfluidic device were used to clamp mother cells in place while daughter cells were washed away by hydrodynamically controlled flow of the surrounding liquid medium. Survival curves are drawn based on data pooled from multiple experiments with accompanying controls; mean RLS and number of mother cells scores are shown for each curve. Survival curves and cell‐cycle curves were plotted using Matlab.

### 2.4 Cell lines and cell culture

Human diploid fibroblast 2BS and WI38 cells, isolated from female fetal lung fibroblast tissue, have been fully characterized previously (Tang, Zhang, Zheng, Corbley, & Tong, 1994). These cell lines were originally established at National Institute of Biological Products. 2BS cell line was kindly granted by professor Tong of Peking University. WI38 was purchased from National Institute of Biological Products. Cells were grown in *Dulbecco's Modification Eagle's Minimum Essential Medium* (DMEM, Life Technologies Inc.) supplemented with 10% fetal bovine serum (FBS; Gibco), 2 mm glutamine, 100 U/ml penicillin, and 100 μg/ml streptomycin, in a humidified atmosphere with 5% CO_2_ at 37°C. These cells were considered as young at PD30 or below and fully senescent at PD50 or above.

### 2.5 Cell cytotoxicity and growth assays

The CCK‐8 assay kit (Dojindo) was used for testing cytotoxicity in vitro. At PD45, 2BS or WI38 cells were seeded into flat‐bottomed 96‐well microplates at a density of 5 × 10^3^ cells/0.2 ml per well. After 20 hr, when the cells reached a subconfluent state, the cells were transferred to a special culture medium containing various concentrations of BBR for further growth, at 37°C in 5% CO_2_ up to 24 hr. Then, the CCK‐8 solution (diluted 0.1 times with 10% FBS DMEM) was added to each well and the cells were incubated for 1 hr at 37°C. The absorbance values of each well were determined spectrophotometrically at 490 nm using a microplate reader (Biotek, MQX200).

Cell viability (%) = (OD _treatment group_ − OD _blank_)/ (OD_control group_ − OD _blank_) × 100.

Cell proliferation was assayed using the CCK‐8 method. Cells were seeded into 96‐well plates at 2.5 × 10^3^ cells/0.2 ml per well and incubated with different concentrations of BBR (0, 0.3125, 1.25, and 5 μg/ml) for one week. The absorbance values of each well were measured at day 0 (4 hr after plating) and on days 1, 2, 3, 4, 5, and 6. The medium containing BBR or DMSO was refreshed every 24 hr. At the indicated points, cells were harvested with 10% CCK‐8 for one hour, and the absorbance was measured at 490 nm. Each data point was measured five times, and each curve was repeated more than three times.

### 2.6 Cell‐cycle analysis

cell‐cycle analysis was conducted according to a previously described procedure (Duan, Zhang, & Tong, 2001). Briefly, WI38 or 2BS cells, grown to approximately 85% confluency at PD45, were split in the ratio of 1:2. One of them was resuspended in DMEM containing different concentrations of BBR (0, 0.3125, 0.625, 1.25, and 2.5 μg/ml) and incubated at 37°C in 5% CO_2_ for 72 hr, while the other one was resuspended in normal DMEM and incubated under similar conditions. The DNA content of the cells was measured by fluorescence‐activated cell sorting method using a Becton‐Dickinson FACScan Flow Cytometry System (BD). The data were analyzed using CellFIT software.

### 2.7 Senescence‐associated β‐galactosidase (SA‐β‐gal) assay

2BS cells (PD45) grown in DMEM with/without different BBR concentrations (0, 0.3125, 0.625, 1.25, and 2.5 μg/ml) were stained according to the method of Dimri with minor modifications (Dimri et al., 1995). Cells grown on plates were washed with PBS, fixed in 4% formaldehyde for 5 min at room temperature, and washed again with PBS. Then, cells were incubated overnight at 37°C without CO_2_ in a freshly prepared staining buffer (1 mg/ml 5‐bromo‐4‐chloro‐3‐indolyl‐β‐d‐galactopyranoside (X‐gal), 40 mm citric acid/ sodium phosphate, pH 6.0, 5 mm potassium ferrocyanide, 5 mm potassium ferricyanide, 150 mm NaCl, and 2 mm MgCl_2_) for 16 hr before being examined according to the instruction manual (CST, 9860S). The average value of the percentage of SA‐β‐gal‐positive cells for each set of samples was calculated based on three independent experiments.

### 2.8 Western blot analysis

Total protein was extracted using RIPA lysis buffer containing protease inhibitor cocktail (Roche). Total protein was measured by BCA (Pierce) assay, and equal amounts of proteins were subjected to 10% SDS‐PAGE and then transferred to polyvinylidene difluoride membranes (Millipore Corp.). The membranes were blocked and then incubated with primary antibodies against ACTIN (ABclonal Technology), P16, Cyclin D1, CDK4, pRB, and RB (ProteinTech) at 4°C overnight with gentle agitation. This was followed by incubation in the presence of goat anti‐rabbit IgG or goat anti‐mouse IgG (Santa Cruz) labeled secondary antibodies at room temperature for 45 min. Each sample was washed thrice in TBS‐T (10 min each) after labeling with the secondary antibody. Blots were developed with an ECL Plus kit (Amersham Biosciences). The images were scanned using an Epson scanning system, and the data were expressed as the values relative to the control. While probing for multiple targets, stripping and re‐probing a single membrane were required. The same membrane was incubated in a stripping solution (Applygen) at room temperature with gentle agitation for 20 min, followed by a 5 min wash in TBS‐T. Then, the membrane was blocked and incubated in the next primary antibody. Data were acquired as detailed above.

### 2.9 *In vivo *experiments

All young and natural aged mice were purchased from SPF Biotechnology Co., Ltd, Beijng, China. C57BL/6J male mice, used as wild‐type (WT) mice (8 weeks/18 months/22 months of age), were fed a normal diet (ND) with free access to water. The mice were kept in an environment with minimum stress along with standard conditions, that is, constant temperature and 12:12 hr light/dark cycle. The animals were allocated at random to a control group and treatment groups. The animals in the treatment group were administered 50 mg/kg of BBR in saline, a dose that was chosen according to a previous study (Zhao et al., 2013). The BBR was administrated orally for four consecutive months (once a day). Animals in the control group were administered the same volume of saline.

An accelerated aging mice model was prepared by administering a common chemotherapeutic drug, doxorubicin, intraperitoneally which can induce senescence in both rodents and humans. The drug was administered at the 10 mg/ml dose, twice weekly.

### 2.10 Rotarod test

Rotarod test was performed using the Rotarod apparatus. Specifically, 23 males (12 WT and 11 BBR treated) before and after BBR treated for four months were trained with three rounds of rotarod tests for 3 days, and at the 4th day, data from three successive experiments were recorded. Final data are the average of the three experiments performed the 4th day (Nobrega‐Pereira et al., 2016). The investigator was blind to the animal grouping.

### 2.11 Statistical analyses

All the experiments were performed at least five times. All results are represented as the mean ± *SEM*. Data involving only two groups were analyzed by one‐ or two‐way ANOVA test. A *p*‐value of < .01 (two stars) was considered as statistically significant.

### 2.12 Ethics statement

All procedures in this study were carried out following the recommendations of the Guide for the Care and Use of Laboratory Animals of China Association for Laboratory Animals Science. All animal care protocols were approved by the Animal Care Committee of Peking University Health Science Center. All sacrifices were performed under pentobarbitone anesthesia, and every effort was made to minimize animal suffering.

## 3 RESULT

### 3.1 Effects of berberine on yeast lifespan

The development of anti‐aging drugs is a tedious and time‐consuming process because the long‐term lifespan experiments need to be performed to achieve meaningful results. *Saccharomyces cerevisiae* (Xie et al., 2015) is one of commonly used model organisms for aging research. The budding yeast *S. cerevisiae* shows progressive aging similar to mammalian dividing cells (Gershon & Gershon, 2000). A microfluidic chip‐based device has been developed to automate the lifespan assay of yeast (Jo, Liu, Gu, Dang, & Qin, 2015), and this technology provides medium‐throughput screening results within three days. Since the aging pathways in yeast and other model organisms are highly conserved, it was proposed to first screen the anti‐aging compounds in yeast before testing them in higher species.

**Figure 1:**
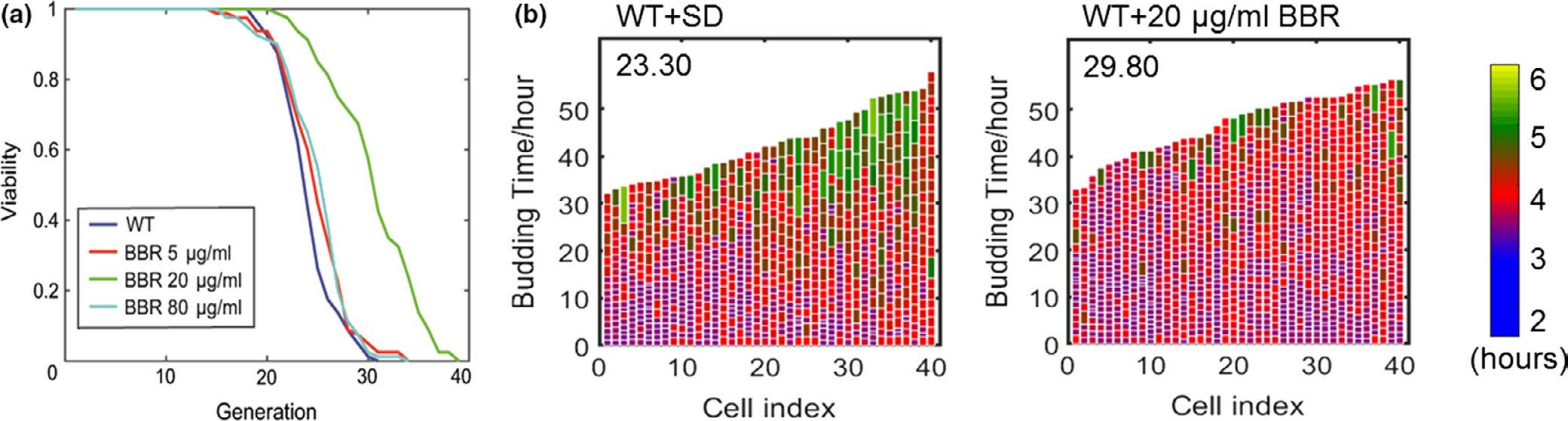
Effects of BBR at low concentrations on the lifespan of yeast. (a) BBR‐treated mother cells showed prolonged replicative lifespan as compared to wild‐type cells in *SD* medium (number of cells [n]: WT, 80; 5 μg/ml, 80; 20 μg/ml, 80; 80 μg/ml, 80. *p‐*value for difference between 20 μg/ml and WT < 0.001). (b) Mother cell budding profiles of wild‐type and BBR‐treated cells (20 μg/ml), showing cell‐cycle duration and heterogeneity (see exponential color scale; cell cycles with durations 1.4hr or less were colored in purple). The x‐axis displays individual mother cells shown as vertical bars, with budding events indicated as horizontal white division. Mean lifespan for each group is presented in the upper‐left corner of the plot

The *SD* media was used in the experiments as yeast has a shorter lifespan in this media as compared to the YEPD media, enabling quicker data acquisition. Cells were taken from solid YEPD media and incubated in the *SD* media for 20 hr and harvested and loaded onto the chip, where the cells were continuously visualized by continuous microscopic imaging for over two days. BBR was added to *SD* media at the concentration of 0, 5, 20, and 80 μg/ml, and the lifespan of yeast in the presence of BBR was evaluated. It was found that BBR at 20μg/ml (≈ 59.46 μm) extends the lifespan of yeast by 28% (Figure 1a). Additionally, the number of cells with long cell‐cycle durations was significantly reduced when treated with 20 μg/ml of BBR (green‐colored cells in Figure 1b). Thus, BBR reduced the heterogeneity of cell‐cycle length, resulting in a longer lifespan (other concentrations are shown in Figure S1a). Therefore, at low concentrations, BBR showed potential anti‐aging effect.

### 3.2 Effects of berberine on cell growth and morphology

**Figure 2:**
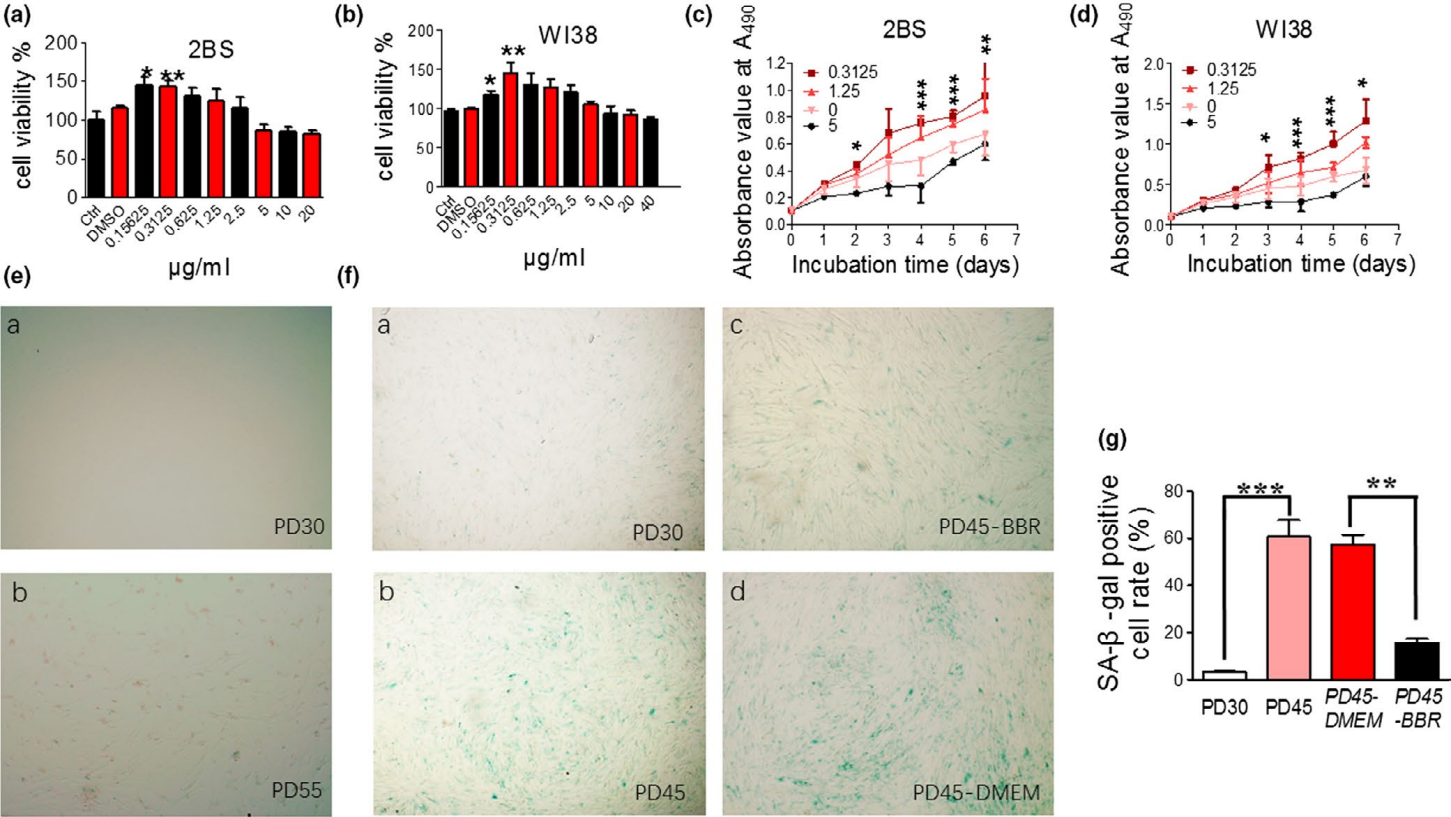
Effects of berberine on cell growth and morphology. (A–B) Effect of BBR at different concentrations on the proliferation of PD45 2BS cells & WI38 cells measured by MTT‐assay. (C–D) Time course of the effect of BBR on proliferation of 2BS & WI38 cells. BBR at 0.3125 ug/ml promoted cell proliferation most. (E) Cell state or morphology of early PD 2BS cells (a) and very late PD 2BS cells (b). (F) SA‐β‐gal staining of early PD 2BS cells (a), late PD 2BS cells (b), late PD 2BS cells grown in DMEM supplemented (c) with or (d) without 0.3125 μg/ml BBR. SA‐β‐gal staining was significantly reduced after BBR treatment. (G) Quantification of SA‐β‐gal‐positive cell rates of 2BS cells. ^***^ *p* < .001 vs. PD30, *n* = 3–5. *^###^p* < .001 vs. PD30, *n* = 3–5. Statistical significance was assessed using one‐way ANOVA test for all experiments. Bar represents the mean ± *SEM*. ^*^ *p* < .05; ^**^ *p* < .01; ^***^ *p* < .001 vs. control, *n* = 3–5

We further studied the effects of low concentration BBR on replicative senescence and its mechanism using human fetal lung diploid fibroblast cells (2BS and WI38). These cell lines are considered as young at PD30 or below and fully senescent at PD55 or above. Cell proliferation was used to evaluate the aging state of the cultured cells. First, the optimum concentration of BBR was determined by evaluating the effects of different concentrations of BBR on growth and proliferation of 2BS (PD45) and WI38 (PD45) cells (Figure 2a,b, respectively). For comparison, the effect on young cells is shown in Figure S1b. The optimum concentration was found to be 0.3125 μg/ml (≈0.929 μm). Above that, BBR (>5 μg/ml ≈ 14.865 μm) inhibited cell proliferation. This result was in agreement with previous studies showing that BBR at concentrations of 10 μm or higher inhibited cell proliferation in a dose‐dependent manner in cancer cells (Sefidabi, Mortazavi, & Hosseini, 2017). However, there are no reports on the effects of low concentrations berberine on cell proliferation. Herein, we observed that berberine, at a concentration of 0.3125 μg/ml, promoted proliferation of 2BS (PD45) and WI38 (PD45) cells during seven days of incubation (Figure 2c,d).

Furthermore, we investigated cell morphology and senescence‐associated markers, such as SA‐β‐gal. Young 2BS cells (PD30) had a flat, spread‐out appearance, with uniform spacing (Figure 2Ea), while the untreated cells in late PD level (PDL) cells showed characteristics of senescence with an accumulation of granular cytoplasmic inclusions (Figure 2Eb). The positive rate of cells for senescence‐associated β‐galactosidase (SA‐β‐gal) is usually used as a marker of population senescence in culture (Dimri et al., 1995). Only 3.5% SA‐β‐gal‐positive 2BS cells were seen in PD30 cells (Figure 2Fa), while 60% were observed in late PDL cells (colored in blue in Figure 2Fb). In BBR‐treated 2BS cells (PD45), the SA‐β‐gal‐positive cell rate reverted to the level of young cells (16%, Figure 2Fc), as compared to the control group (56.5%, Figure 2Fd). Quantification analysis is shown in Figure 2g. These results suggest that BBR either delayed or reversed the population senescence of 2BS and WI38 cells. However, at late PD the senescent phenotype of the cells could be alleviated but not reversed completely.

### 3.3 Berberine promotes G1/S transition in aged cells

**Figure 3:**
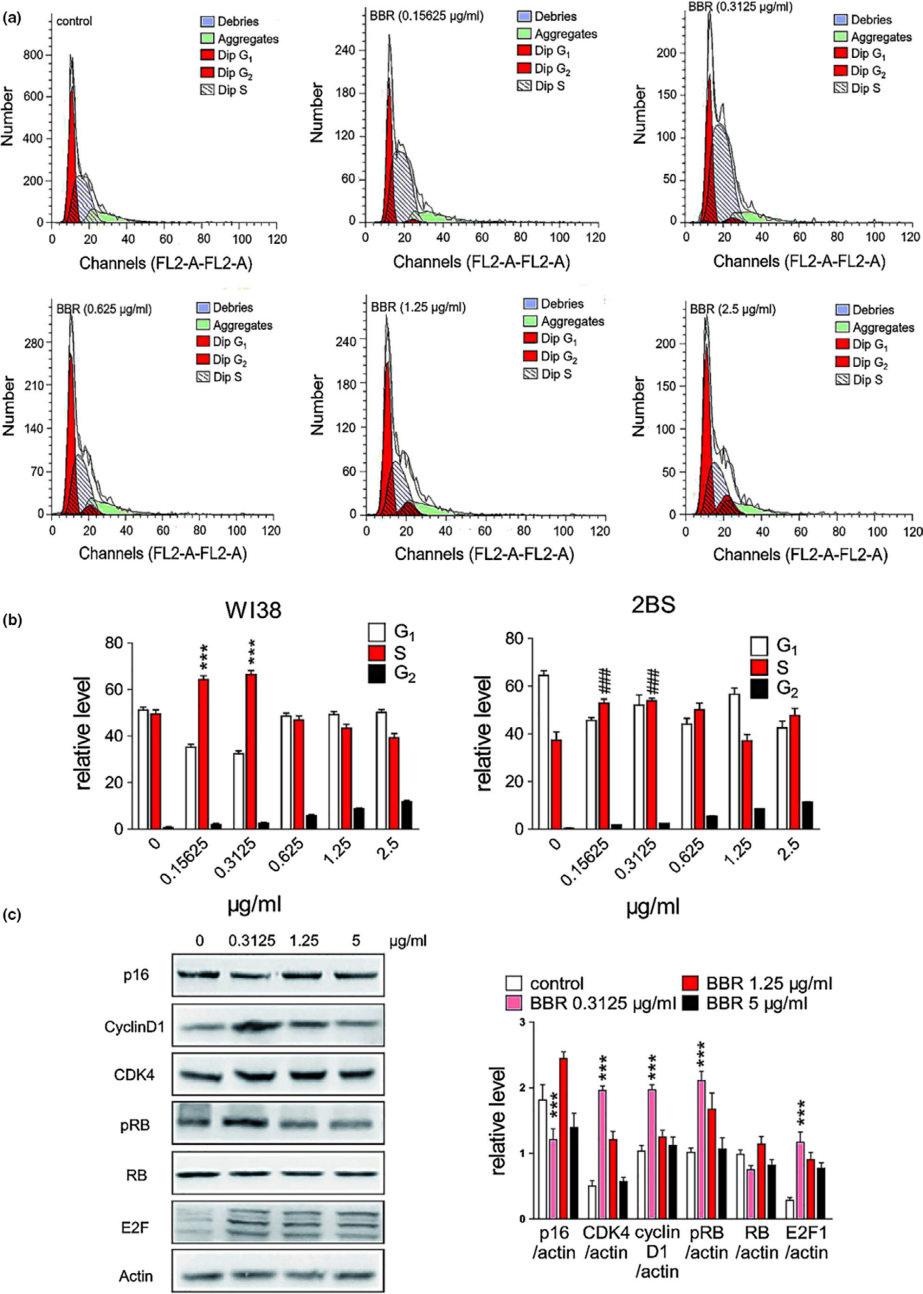
Effect of BBR on cell cycle and cell cycle‐related proteins. (a) Phase analysis of cell cycle in WI38 cells and BBR‐treated cells by flow cytometry. The fraction of S phase cells reached maximum at 0.3125 μg/ml of BBR. (b) Abundance of different cell‐cycle phases of WI38 & 2BS cells on different BBR concentration. ^***^ *p* < .001 vs. WI38 control, *n* = 3–5. *^###^p* < .001 vs. 2BS control, *n* = 3–5. G_1_‐S phase transition was observed statistically from 0 to 0.3125 μg/ml. (c) Changes in the expression of p16, cycline D1, CDK4, pRB, RB, and E2F as determined by Western blot. Statistical significance was assessed using the two‐way ANOVA test for all experiments. Bar represents the mean ± *SEM*. ^*^ *p* < .05; ^**^ *p* < .01; ^***^ *p* < .001 vs. control, *n* = 3–5

To understand the mechanism involved in delayed senescence by BBR, we evaluated the level of markers related to replicative senescence and the cell‐cycle profiles. The WI38 (PD45) and 2BS (PD45) cell‐cycle profiles were analyzed using flow cytometry (Figure 3a and Figure S2). The percentage of WI38 (PD45) and 2BS (PD45) cells in G_1_/S/G_2_‐M phase without/with BBR at different concentrations were quantified (Figure 3b). The percentage of cells in S/G_2_‐M phase at 0.3125 μg/ml of BBR were higher by ~18% than at other concentrations, indicating that low concentration of BBR (0.3125 μg/ml) could promote the entry of aged WI38/2BS cells from G_0_ or G_1_ phase to S/G_2_‐M phase. This was consistent with earlier findings in the yeast cells.

Next, we evaluated the levels of p16, cyclin D, CDK4, cyclin E, RB/pRB, and E2F proteins in WI38 (55PD) cells using Western blot (Figure 3c). When cells were treated with BBR (0.3125 μg/ml), the level of p16 decreased and that of cyclin protein and cyclin‐dependent kinases, such as cyclin D1 and CDK4, increased. Meanwhile, the level of phosphorylated retinoblastoma protein (pRB), which plays an important role in anti‐aging, was up‐regulated. The retinoblastoma protein (RB) is a tumor suppressor protein that prevents excessive cell growth by inhibiting cell‐cycle progression until a cell is ready to divide. Phosphorylation of RB to pRB induces the release of E2F1 that promotes the synthesis of DNA and allows cell‐cycle progression. In fact, the cyclin D1 interacts with its specific kinases (CDK4 and/or CDK6) resulting in cell‐cycle development through RB phosphorylation (Bienvenu et al., 2010). Previous studies have illustrated that cyclinD1/CDK4 interaction during G_1_/S phase results in a complex formation which consequently regulates the proliferation of aging cells (Beumer, Roepers‐Gajadien, Gademan, Kal, & Rooij, 2000). Hence, any disruption in cyclin D1 and CDK4 expressions would remarkably impact the cell‐cycle arrest during cellular proliferation. Together, this experimental data indicated that BBR ameliorated the replicative cellular senescence by up‐regulating cyclin D1 and CDK4 expressions.

### 3.4 Berberine counteracts doxorubicin‐induced chemotoxicity in vitro and in vivo

**Figure 4:**
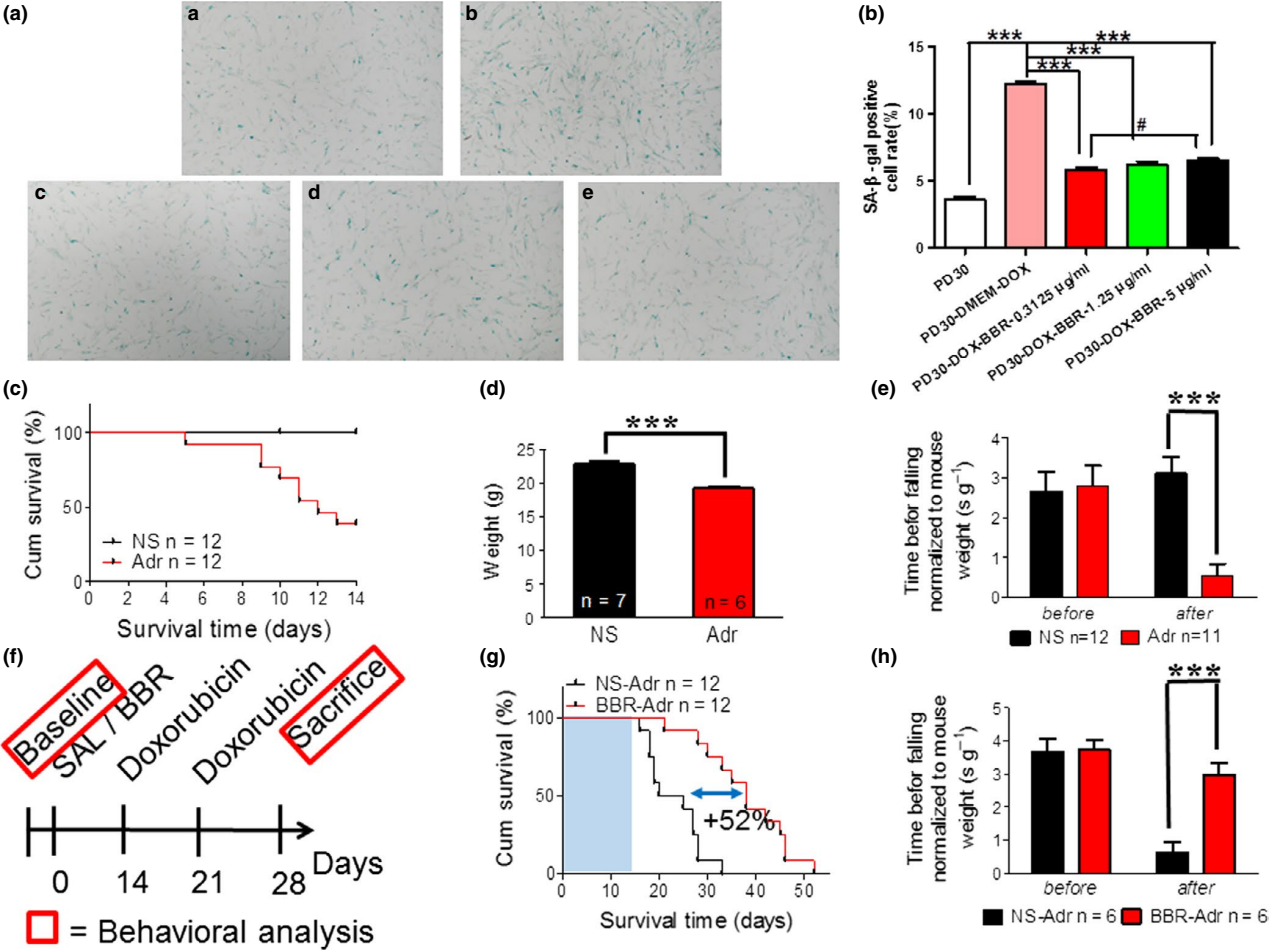
Berberine counteracts doxorubicin‐induced senescence and chemotoxicity in vitro and in vivo. (A) SA‐β‐gal staining assay in early PD (P30) WI38 cells (a), doxorubicin‐induced P30 WI38 cells (b), BBR (0.3125 μg/ml (c), 1.25 μg/ml (d), 5 μg/ml (e)) treated doxorubicin‐induced P30 WI38 cells. (B) Quantification of SA‐β‐gal‐positive cell rates of WI38 cells in (A). ***p < .001, *n* = 3–5. *^#^p* < .05, *n* = 3–5. (C) Survival curve of mice treated with 10 mg/kg doxorubicin (*p‐*value < .001 using log‐rank test). (D) The change in body weight in mice treated with 10 mg/kg doxorubicin. (E) Rotarod test on doxorubicin accelerated aging mice. (F) Schematic timeline of experiments in (G–H). Doxorubicin: twice i.p. at dose of 10 mg/kg. (G) Survival curve of accelerated aging mice treated with 100 mg/kg BBR first (*p‐*value < .0001 using log‐rank test). (H) Rotarod test on accelerated aging mice treated with 100 mg/kg BBR first. Statistics significance was assessed using the two‐way ANOVA test for all experiments. Bar represents the mean ± *SEM*. ^*^ *p* < .05; ^**^ *p* < .01; ^***^ *p* < .001 vs. control, *n* = 3–5

Off‐target toxicity limits the maximum tolerated dose of chemotherapeutic drugs and causes long‐term health problems in cancer survivors, including accelerated aging (Henderson, Ness, & Cohen, 2014). Since chemotherapy can induce senescence (Ewald, Desotelle, Wilding, & Jarrard, 2010), we explored whether BBR could rescue cells after chemotherapy. Doxorubicin (Dox), a chemotherapeutic drug known to induce senescence (Cahu, Bustany, & Sola, 2012) and liver toxicity in rodents and humans (Damodar, Smitha, Gopinath, Vijayakumar, & Rao, 2014), was used as a model drug. Dox (0.1 μm) was used to induce senescence in WI‐38 in vitro, evident by elevated SA‐β‐GAL activity. Then, we tested whether BBR could affect premature, stress‐induced cellular senescence caused by Dox. The accelerated aged cell model samples were treated with 0.3125, 1.25, and 5 μg/ml BBR after Dox addition followed by evaluation of the level of senescence using SA‐β‐gal. We found BBR was able to rejuvenate premature senescence in Dox‐treated WI38 cells in a dose‐dependent manner (Figure 4a,b). Next, we performed in vivo experiments at 10 mg/kg dose in mice. Treatment with Dox reduced the total body weight and exercise performance as well as lifespan in 8‐week‐old mice (Figure 4c–e). Next, we treated the mice with both BBR (100 mg/kg) and Dox through a procedure as shown in Figure 4f. The effective dose of BBR used in these experiments was chosen based on a previous study (Mehrzadi et al., 2018). We used slightly lower dose because of longer treatment period. Strikingly, the median lifespan of the treatment group was extended by ~52% (Figure 4g) and the coordination measured based on retention time on rotarod of the BBR group was significantly longer than those of the control group (Figure 4h), indicating better physical condition. Taken together, BBR was effective in reducing doxorubicin‐induced senescence in vitro and alleviated the doxorubicin‐induced loss in body weight and the reduction in coordination. Thus, BBR may be used to treat cytotoxicity resulting from chemotherapeutic drugs.

### 3.5 Berberine prolongs the lifespan of naturally aged mice

**Figure 5:**
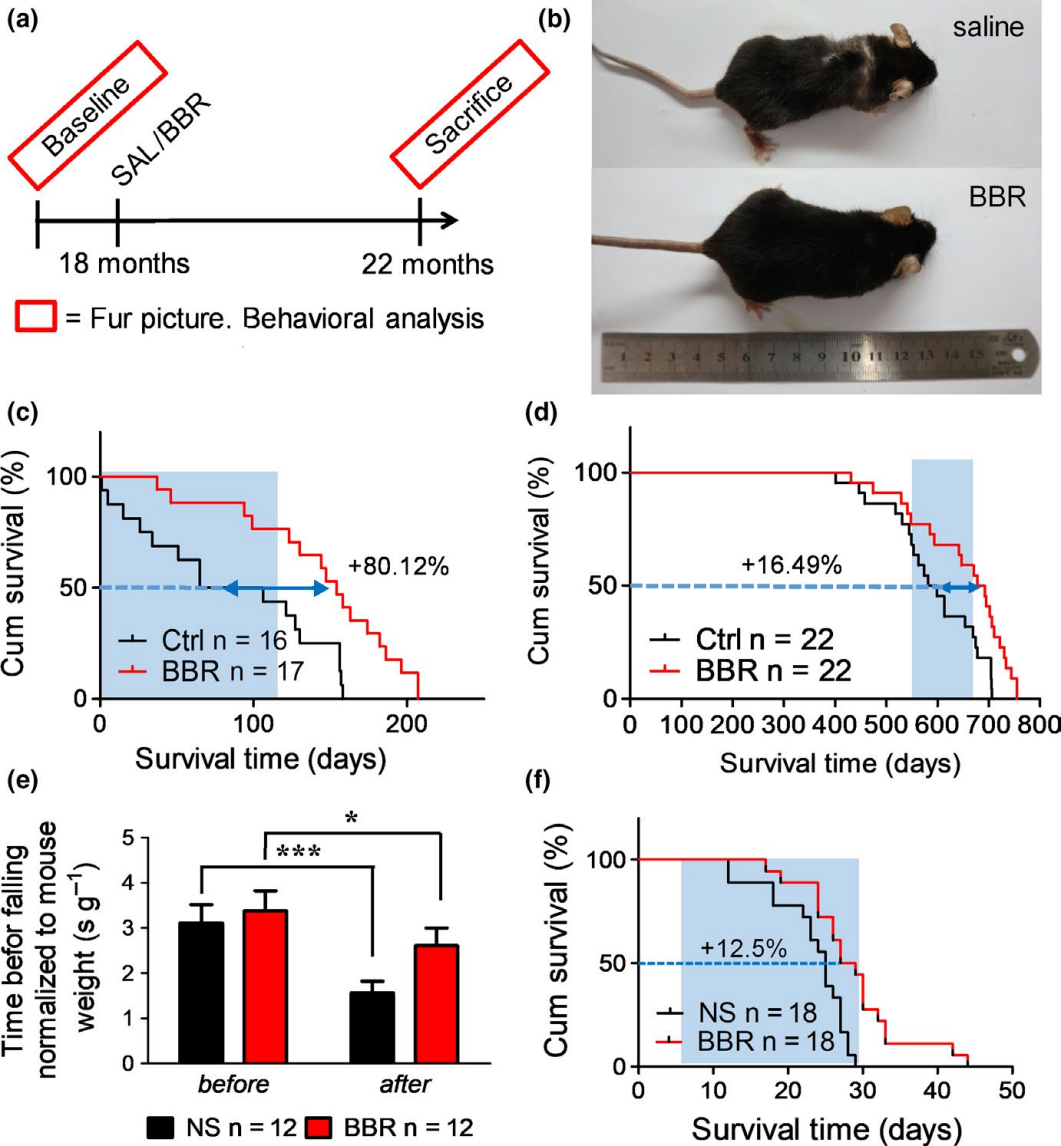
Effects of berberine administration on lifespan, body weight, and exercise performance in naturally aged mice. (a) Schematic timeline of the experimental procedure of BBR on 18 months naturally aged mice (C57BL/6J male). (b) The picture of 18 months naturally aged mice treated with 50 mg/kg BBR for 4 months showing difference of fur density. (c) Survival curve of 18 months naturally aged mice treated with 50 mg/kg BBR. Blue shade indicates the treatment window (*p‐*value < .0025 using log‐rank test). (d) Entire lifespan of 18 months naturally aged mice treated with 50 mg/kg BBR. Blue shade indicates the treatment window (*p‐*value < .007 using log‐rank test). (e) Rotarod test on 18 months naturally aged mice treated with 50 mg/kg BBR for 4 months. (f) Survival curve of 22 months naturally aged mice treated with 50 mg/kg BBR (*p‐*value < .0022 using log‐rank test)

Based on these results, we expected BBR to extend the lifespan in naturally aged mice. The procedures we followed are shown in Figure 5a. Two independent experiments were conducted, one using 18‐month‐old mice (18M) and the other using 22‐month‐old mice (22M). We further reduced the dose to 50 mg/kg because of even longer treatment period. For the 18M and 22M groups, BBR was administered for four months and one month, respectively. The survival curves were recorded, and coordination was tested for all control and treated mice. Additionally, photographs of the 18M group mice were taken before sacrifice (˃20 months) to record the difference in fur density, a characteristic feature of aging in mice (Figure 5b and Figure S3 b). We observed that the mice from the control group (on the right) had sparse furs, especially on the back of the body and had less glossy hair as compared to BBR group. Administration of BBR extended the median residual lifespan from 85.5 days to 154 days (68.5 days, ~80% extension) as shown in Figure 5c. The entire lifespan was extended by ~ 16.49% as shown in Figure 5d. The coordination in the treatment group was also well maintained (Figure 5e). Furthermore, there was no significant difference in weekly average body weight between control and treatment groups indicating the safety of regular administration of relatively low doses of BBR (Figure S3 c). The mice from the 22M group were too old for the coordination test. Therefore, only survival curves of mice from the 22M group were recorded from the day when BBR was administered. BBR extended the median residual lifespan by ~ 12.5% (Figure 5f). Thus, we found that BBR has anti‐aging effect in naturally aged mice.

## 4 DISCUSSION

With an increase in life expectancy in the foreseeable future, it is important to develop strategies to extend both the lifespan and health span in the aging population. Herein, BBR was evaluated for its anti‐aging effects at relatively low concentration in vitro and in vivo. As aging is a multi‐facet process, an anti‐aging drug is likely to be related to multiple processes. Our findings would co‐exist with known mechanism of BBR like mTOR pathway, reducing DNA damage responses (Zhao et al., 2013). In fact, cell proliferation might be the last step for showing the anti‐aging phenotype. Thus, the cell‐cycle part of the mechanism might be the last piece of the puzzle. Specifically, we found that BBR down‐regulated p16 and up‐regulated cyclin D1 and CDK4 expression, promoting cell‐cycle progression. Additionally, we observed the protective effect of BBR against aging could be related to its effect on the expression of p16, RB, cyclin protein, and their specific kinases.

Cyclins, the regulatory subunits of cyclin‐dependent kinases (CDKs), control the phase transition through core checkpoints in the cell cycle. Major regulatory pathways leading to proliferation occur in the G_1_ phase of the cell cycle. The core controlling machinery of cell‐cycle monitors chromosomal DNA duplication, integrity, replication, and repair. After these controls, the machinery causes cell transition from G_1_ to S phase. Indeed, previous studies have revealed that suppression of cyclins expression and/ or synthesis negatively affects cell growth and proliferation (Bloom & Cross, 2007). Three different cyclins, D1, D2, and D3, have been identified in various mammalian cells. Cyclin D1 is mainly involved in the cell‐cycle process; it plays critical roles in G_1_/S transition. It has been identified that cyclin D1 plays two opposing roles including cell proliferation and cell‐cycle arrest: DNA damage causes rapid elimination and reduction in cyclin D1 expression and then activation of cyclin E‐CDK2 pathway through the degradation of cyclin D1 by the proteasome (Bienvenu et al., 2010).

In addition to cyclins, p16 is another key factor known to participate in cyclin‐related cell‐cycle machinery. p16 is a member of the cyclin kinase inhibitor family that inhibits CDK complex formation resulting in cell‐cycle arrest and apoptosis (Li et al., 2013). Aging‐induced intensive DNA damage triggers overexpression of p16, which in turn inhibits cyclin D1 and CDK4 complex formation and induces cell‐cycle arrest after DNA fragmentation.

This study revealed that severe DNA damage in accelerated or natural aging models was ameliorated by BBR both in vitro and in vivo. In fact, BBR down‐regulated the expression of p16, which promoted the formation of the cyclinD1‐CDK4 complex. It is noteworthy that the mechanism by which BBR modulates p16 is still unknown. Further study needs to be carried out to make it clear. This was followed by phosphorylation of RB into pRB by the cyclinD1‐CDK4 complex. As a result, pRB induced the release of E2F1 which promoted the synthesis of DNA and allowed cell‐cycle progression. Therefore, BBR ameliorates cellular senescence by promoting cellular transition from G1 to S phase during early mitosis of the cell cycle.

However, BBR is a natural product that has poor drug‐like properties. Although it could be absorbed in the gastrointestinal tract after oral administration, its pharmacokinetic properties (absorption, tissue distribution, metabolism, and elimination) are poor and it has low plasma concentration and low absolute bioactivity (<1%) (Jin, Khadka, & Cho, 2016). A 5 mg/kg dose yields 1.7 μg/ml peak plasma concentration (Wang et al., 2005). Furthermore, BBR undergoes extensive metabolism after oral administration and absorbed BBR is converted into multiple metabolites, resulting in more than 20 BBR‐related metabolites such as berberrubine, Jatrorrhizine, demethyleneberberine, and their corresponding glucuronides (Wang, Feng, Chai, Cao, & Qiu, 2017). Moreover, the route of administration is an important factor that affects the toxicity of BBR. The median lethal dose (LD50) of BBR in mice after intravenous injection and intraperitoneal injection was calculated as 9.03 and 57.6 mg/kg, respectively. However, no LD50 was found for the oral administration group, which indicates that the acute toxicity of BBR was related to the type of administration route.

It was found that 20.8g BBR per kg of body weight is safe for oral administration in mice, while the safe dose for humans would be 2.97 g BBR per kg of body weight. The difference in safe dosage may be attributed to the mice having a 7‐fold higher metabolic rate per kg body weight than adult humans. To date, no serious adverse effects have been reported for BBR administration via oral route in clinic, and it is shown to be safe in the majority of human subjects studied during short‐term and long‐term use (Liu, Zheng, Zhang, & Long, 2016). Although BBR is a safe and effective natural product, the pharmacokinetic studies in rodents and humans have consistently concluded that BBR has poor systematic bioavailability after oral administration. Its poor absorption in the gut and rapid metabolism in the body are two major obstacles for the effective use of BBR as a drug.

One possible concern about BBR would be if it increases the risk of cancer by promoting cell proliferation. Though we showed BBR promotes cell proliferation in WI38, it is reported previously BBR treatment promotes cell‐cycle arrest and death in human cancer cell lines, coupled to an increased expression of apoptotic factors (Sefidabi et al., 2017). BBR was reported to leading to apoptosis in around 20 types of cancers through various mechanisms. Thus, BBR is evident to rejuvenate aging cells and inhibit cancer cells at the same time.

A complication of this study is that mice we used have a median lifespan around 600 days for both 18M and 22M groups. It is 100–200 days shorter than that from other laboratories. For the 22M group, half of the whole group has been dead when we took over; thus, the difference comes from the strain itself rather than our treatment. Since these mice may be considered to be short‐lived mice with unknown inducer, we cannot fully exclude the possibility that BBR just corrects it. As the mice did not show any unusual phenotypes, we believe it bares much greater chance to represent the true effect in natural aging process.

Thus, the use of BBR at low doses may result in restoring the loss of health due to natural aging. Therefore, this molecule may be a candidate as an anti‐aging drug for the treatment of age‐related diseases that are, at least in part, driven by senescence.

## CONFLICT OF INTEREST

None declared.
