# Supplementary figures and images for: Berberine ameliorates cellular senescence and extends the lifespan of mice via regulating p16 and cyclin protein expression
Source: Aging Cell. 2019 Nov 26;19(1):e13060. doi: 10.1111/acel.13060 (PMC6974710; doi:10.1111/acel.13060)

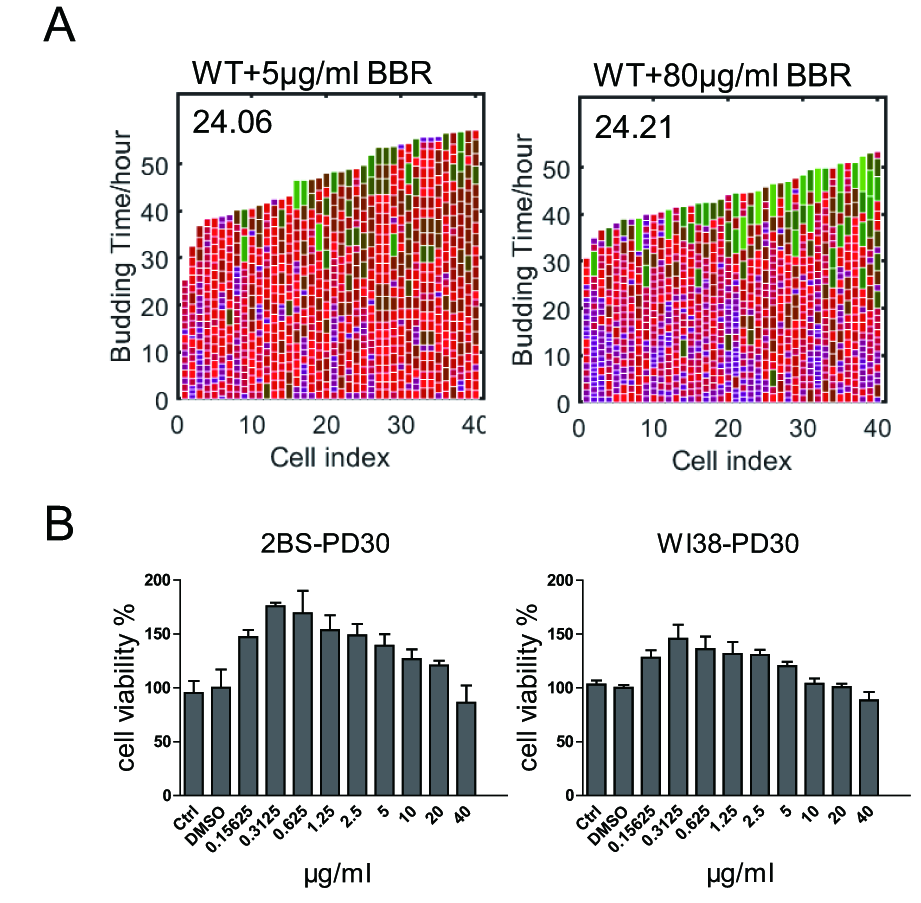

Supplement: Supplementary file 1 [file ACEL-19-e13060-s001.tif]

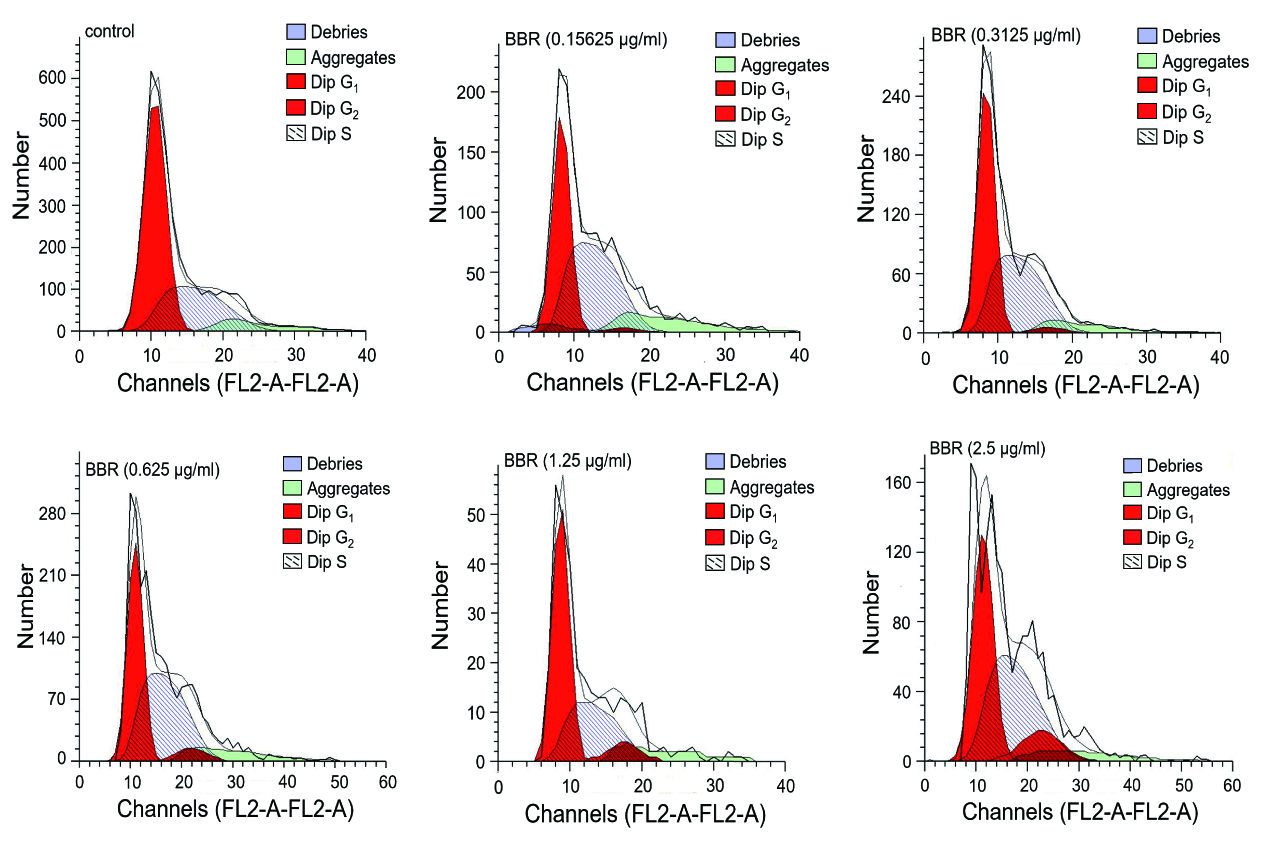

Supplement: Supplementary file 2 [file ACEL-19-e13060-s002.tif]

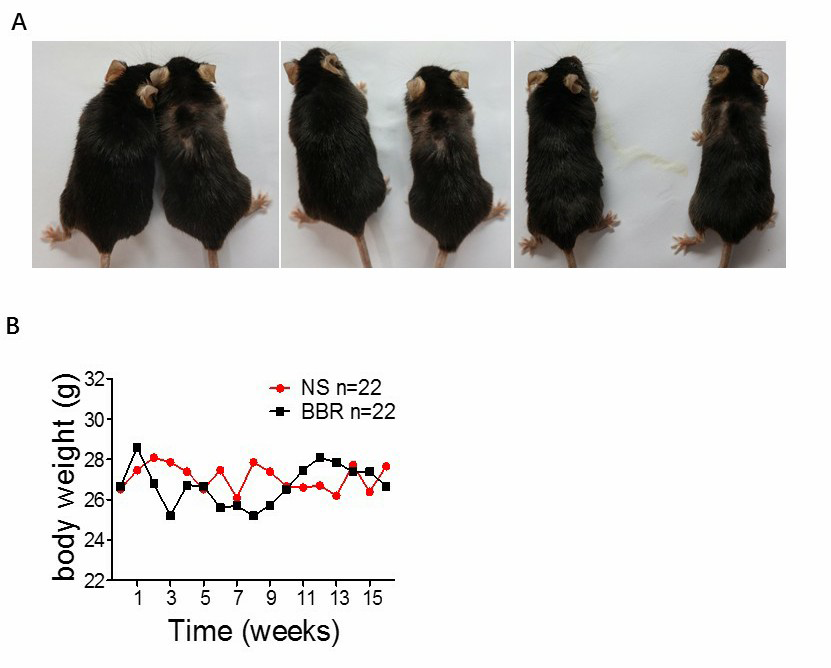

Supplement: Supplementary file 3 [file ACEL-19-e13060-s003.tif]
